# Effects of Temperature on the Pharmacokinetics, Tissue Residues, and Withdrawal Times of Doxycycline in Rainbow Trout (*Oncorhynchus mykiss*) following Oral Administration

**DOI:** 10.3390/vetsci10060401

**Published:** 2023-06-18

**Authors:** Orhan Corum, Kamil Uney, Ertugrul Terzi, Duygu Durna Corum, Devran Coskun, Feray Altan, Muammer Elmas

**Affiliations:** 1 Department of Pharmacology and Toxicology, Faculty of Veterinary Medicine, University of Hatay Mustafa Kemal, Antakya 31060, Türkiye; ddurnacorum@gmail.com; 2 Department of Pharmacology and Toxicology, Faculty of Veterinary Medicine, University of Selcuk, Konya 42031, Türkiye; kuney@selcuk.edu.tr (K.U.);; 3 Faculty of Fisheries, University of Kastamonu, Kastamonu 37200, Türkiye; ertugrulterzi@gmail.com; 4 Department of Pharmacology and Toxicology, Faculty of Veterinary Medicine, University of Siirt, Siirt 56100, Türkiye; devrancoskun@gmail.com; 5 Department of Pharmacology and Toxicology, Faculty of Veterinary Medicine, University of Dokuz Eylul, Izmir 35140, Türkiye; altanferay@gmail.com

**Keywords:** doxycycline, oral pharmacokinetics, rainbow trout, temperature, tissue residue, withdrawal time

## Abstract

**Simple Summary:**

Doxycycline, an approved aquacultural antibiotic, is extensively used in the treatment of bacterial diseases in fish. Since fish are poikilothermic organisms, their body temperature and metabolic rate are primarily influenced by the temperature of the water. Therefore, temperature may be affected by pharmacokinetic behavior and withdrawal times of drugs. The current study was undertaken to look at the differences in pharmacokinetics, tissue residues, and withdrawal times of doxycycline following oral administration in rainbow trout reared at 10 and 17 °C. The increment of water temperature from 10 to 17 °C decreased the elimination half-life, the body clearance, and the distribution volume of doxycycline and increased plasma concentrations. The withdrawal times for plasma and tissues decreased with the temperature increase. The results contributed to the determination of an optimal dosing regimen and the safe consumption of edible tissues in rainbow trout that were administered doxycycline and reared at different temperatures.

**Abstract:**

The purpose of this study was to compare the pharmacokinetics, tissue residues, and withdrawal times of doxycycline after oral administration in rainbow trout reared at 10 and 17 °C. Fish received a 20 mg/kg oral dose of doxycycline after a single or 5-day administration. Six rainbow trout were used at each sampling time point for plasma and tissue samples, including liver, kidney, and muscle and skin. The doxycycline concentration in the samples was determined using high-performance liquid chromatography with ultraviolet detector. The pharmacokinetic data were evaluated by non-compartmental kinetic analysis. The WT 1.4 software program was used to estimate the withdrawal times. The increase of temperature from 10 to 17 °C shortened the elimination half-life from 41.72 to 28.87 h, increased the area under the concentration–time curve from 173.23 to 240.96 h * μg/mL, and increased the peak plasma concentration from 3.48 to 5.50 μg/mL. At 10 and 17 °C, the doxycycline concentration was obtained in liver > kidney > plasma > muscle and skin. According to the MRL values stated for muscle and skin in Europe and China (100 μg/kg) and in Japan (50 μg/kg), the withdrawal times of doxycycline at 10 and 17 °C were 35 and 31 days, respectively, for Europe and China and 43 and 35 days, respectively, for Japan. Since temperature significantly affected pharmacokinetic behavior and withdrawal times of doxycycline in rainbow trout, temperature-dependent dosing regimens and withdrawal times of doxycycline might be necessary.

## 1. Introduction

Fish is important for human health because it contains high levels of protein, unsaturated fatty acids, and essential nutrients and is easier to digest than other meats [1]. Fish consumption per capita was reported to have increased from 9.9 kg in the 1960s to 19.2 kg in 2012 [2]. Aquaculture has become a large sector as people increase their fish consumption to eat more healthily [3]. Although this growth in the sector has increased economic gain, stress and bacterial infections have become widespread in fish [4]. Therefore, antibiotics are commonly used in the treatment of bacterial infections in fish [3]. In order to protect human health, maximum residue limit (MRL) values of antibiotics have been determined in animals with nutritional value, such as fish [5]. If the drug concentration in edible tissues is higher than the MRL value, it may cause adverse effects such as allergies, diarrhea, vomiting, and hormone-like reactions in humans [6]. In addition, excessive and inappropriate use of antibiotics causes antimicrobial resistance [7]. Therefore, it is essential to examine the pharmacokinetic, tissue residue, and withdrawal time information of antibiotics in the target fish species.

Rainbow trout is one of the most cultivated fish species in Türkiye [8]. Türkiye accounts for approximately 78% of rainbow trout production in the EU and 15% of rainbow trout production in the world [9]. Gram-negative and Gram-positive bacteria such as *Aeromonas hydrophila*, *Yersinia ruckeri*, *Edwardsiella tarda*, *Lactococcus garvieae*, *Vibrio* spp., and *Streptococcus* spp. cause many diseases that cause economic losses and deaths in rainbow trout such as enteric redmouth disease, hemorrhagic septicemia, meningoencephalitis, and exophthalmos [7,10].

Doxycycline is a semi-synthetic oxytetracycline derivative with a broad spectrum of action, including Gram-positive and Gram-negative bacteria, *mycoplasma*, *chlamydia*, *rickettsia*, and protozoan parasites [11]. Doxycycline has a bacteriostatic effect by inhibiting protein synthesis in bacteria [12]. In addition, doxycycline possesses antineoplastic, anti-inflammatory, neuroprotective, and matrix metalloproteinase-inhibiting properties [11]. Doxycycline has several advantages over older analogues, including low affinity for calcium and magnesium, good oral absorption, greater volume of distribution, and tissue penetration due to its lipophilic structure and a long elimination half-life [13,14]. Doxycycline has been widely utilized in the treatment of bacterial diseases in fish due to its low cost and has little adverse effects. Doxycycline is recommended to be used at a 20 mg/kg dose for 3–5 days in infections caused by bacteria such as *Aeromonas hydrophila*, *Edwardsiella ictalurid*, *Vibrio vulnificus*, *Pseudomonas fluorescens*, and *Fibrobacter columnaris* in some fish species in China, Japan, India, and the Philippines [15,16]. The MRL of doxycycline for muscle and skin in fish was 100 μg/kg in Europe and China and 50 μg/kg in Japan [17,18].

Since fish are poikilothermic organisms, their body temperatures and metabolic rates are primarily influenced by the temperature of the water [19]. Türkiye, which has a large geographical area, has four distinct seasons and the temperature of the water varies substantially between areas and seasons. Temperature influences drug absorption, distribution, biotransformation, and excretion in fish [20]. Furthermore, temperature has been shown in some studies to alter the pharmacokinetics of drugs such as florfenicol, enrofloxacin, flumequine, oxolinic acid, and oxytetracycline in fish [19,21]. Temperature has been shown to affect the pharmacokinetics of doxycycline after oral administration in grass carp [19]. Doxycycline can be used to treat bacterial infections in rainbow trout due to its pharmacokinetic and pharmacodynamic features. The effect of temperature on the pharmacokinetics and tissue residues of doxycycline in rainbow trout has not been reported to the best of the authors’ knowledge. This study investigated the pharmacokinetics and tissue residues of doxycycline in rainbow trout at 10 and 17 °C. The goals of this study on rainbow trout at different temperatures were to: (1) investigate the pharmacokinetics of doxycycline after single-dose oral administrations and (2) establish tissue residues and withdrawal time of doxycycline following daily oral administration for five days.

## 2. Materials and Methods

### 2.1. Chemicals

The analytical standard of doxycycline hyclate (>97%) was purchased from TCI (Tokyo Chemical Industry, Tokyo, Japan). All chemicals were provided by VWR International (Fontenay-sous-Bois, France). The acetonitrile was of a purity for high pressure liquid chromatography (HPLC) grade. Ultra-distilled water was used to prepare the solutions.

### 2.2. Animals

Four hundred and twenty healthy rainbow trout (*Oncorhynchus mykiss*), weighing between 150 and 200 g, were procured from a commercial fish farm (Kastamonu, Türkiye). Before being included in the study, the fish were visually inspected for poor body condition, trauma, or signs of disease. The fish were placed in the aquarium recirculation system at random; each aquarium had 100 L of aerated tap water and six fish. The reserve tank water flow rate was 30 L/h and the tank was constantly filled with water from tap water. The water temperature was maintained at either 10 ± 0.5 °C or 17 ± 0.5 °C using an aquarium heater. The following conditions were maintained for water quality parameters: pH at 7.6 ± 0.4, dissolved oxygen levels at 8.10–8.50 mg/L, nitrite levels ˂ 35 μg/L, and concentrations of unionized ammonia 44.4 ± 3.4 ng/L. All fish were acclimated for at least seven days and fed a drug-free trout pellet diet daily. Fish were not fed for 6 h before and after doxycycline administration. The Kastamonu University Animal Experiments Local Ethics Committee (Kastamonu, Türkiye) approved (2020/22) all experimental procedures.

### 2.3. Experimental Design

Doxycycline hyclate was dissolved in purified water at a concentration of 10 mg/mL and administered to the fish. Oral administration was performed by gastric gavage to administer the exact amount of doxycycline according to the body weight of the fish. Drug administration and sampling were performed under anesthesia of MS-222 (tricaine methanesulfonate, 200 mg/L) to gently sedate and reduce stress for animal welfare purposes. Blood samples (approximately 1 mL) were drawn from the caudal vessel using a 26-G needle affixed to a 1 mL syringe and then placed in heparin tubes. Four hundred and twenty rainbow trout were separated into two temperature-based groups: one at 10 °C (n = 210) and the other at 17 °C (n = 210). This investigation was conducted in two experimental designs—pharmacokinetics and residues—at both temperatures. During the pharmacokinetic design (n = 102 for 10 °C, n = 102 for 17 °C), a single dose of 20 mg/kg doxycycline was administered to the fish; blood samples were taken prior to drug administration (0 h) and at 0.083, 0.25, 0.5, 1, 2, 4, 8, 12, 24, 48, 72, 96, 120, 144, 192, and 240 h after drug administration. During the residue design (n = 108 for 10 °C, n = 108 for 17 °C), doxycycline was administered to the fish at a dose of 20 mg/kg every 24 h for 5 days; blood and tissue samples (muscle and skin, liver, kidney) were collected prior to drug administration (0 day) and at 0.04, 0.5, 1, 2, 3, 4, 5, 6, 8, 10, 15, 20, 25, 30, 35, 40, and 50 days after drug administration. Plasma samples were obtained after blood samples were centrifuged at 4000× *g* for 10 min. Until analysis, all samples were kept at −80 °C.

### 2.4. Analytical Method

The plasma and tissue concentrations of doxycycline were assayed by HPLC with ultraviolet detection (UV) using a previous published method [22,23]. Tissues were homogenized for 30 s at 9500 rpm using a tissue homogenizer (Heidolph Silent Crusher M, Schwabach Germany). Then, 100 μg tissue and 100 μL plasma were mixed with 200 μL of buffer/EDTA (0.1 M disodium EDTA, containing 0.1 M sodium phosphate) and 50 μL of perchloric acid (60%). Following a 40 s vortex, the mixture was centrifuged at 15,000× *g* for 15 min and the supernatant was transferred to autosampler vials. The supernatant was injected in an amount of 50 μL onto an Inertsil column (ODS-3, 4.6 mm × 250 mm; 5 μm; GL Sciences, Tokyo, Japan), which was maintained at 40 °C. At a flow rate of 1 mL/min, the mobile phase consisted of 30% acetonitrile and 70% trifluoroacetic acid (0.01 M). The concentration of doxycycline was determined using an HPLC system (Shimadzu, Tokyo, Japan), comprising a degasser (DGU-20A), a column oven (CTO-10A), an auto-sampler (SIL 20A), a pump (LC-20AT controlled by the CBM-20A), and a UV–VIS detector (SPD-20A) with absorbance set at 350 nm.

According to European Medicines Agency regulations, the chromatographic procedure was validated [24]. A stock solution of doxycycline hyclate with a concentration of 1 mg/mL was prepared in purified water. By further diluting the stock solution, working standard solutions of varying concentrations (0.04, 0.1, 0.2, 0.4, 1, 2, 4, 10, and 20 μg/mL) were obtained. Doxycycline calibration standards were linear (R2 > 0.9990) for plasma (0.04, 0.1, 0.2, 0.4, 1, 2, 4, 10, and 20 μg/mL) and tissues (0.04, 0.1, 0.2, 0.4, 1, 2, 4, 10, 20, 40, and 100 μg/g). The plasma and the tissue quality control samples (0.1, 1, and 10 μg/mL) were analyzed in 5 replicates within 5 days to assess recovery, precision, and accuracy. Doxycycline recovery rates were >92% in plasma and >87% in tissues. The lower limit of quantification was 0.04 μg/mL (g) for doxycycline in rainbow trout plasma and tissues with the bias of ±15% and the coefficient of variation <20%. The coefficients of variance for intra-day and inter-day were ≤7.4% and ≤8.6%, respectively. The intra-day and inter-day bias were ±8.0% and ±9.4%, respectively.

### 2.5. Pharmacokinetic Analysis

Using the WinNonlin 6.1.0.173 software program (Pharsight Corporation, Scientific Consulting Inc., Sunnyvale, NC, USA), the plasma concentration–time curves of doxycycline were plotted. After oral administration, pharmacokinetic parameters were determined via non-compartmental analysis using mean plasma concentrations of doxycycline collected at each sampling time [25,26,27]. After the doxycycline administration, apparent volume of distribution (V_darea_/F), total body clearance (CL/F, area under the concentration–time curve (AUC), terminal elimination half-life (t_1/2ʎz_), AUC extrapolated from t_last_ to ∞ in % of the total AUC (AUC_extrap_ %), and mean residence time (MRT) were determined. The peak concentration (C_max_) and the time to reach C_max_ (T_max_) were calculated from the observed data on the plasma concentration–time curve.

### 2.6. Withdrawal Time Analysis

The European Medicines Agency’s WT 1.4 software program was used to determine the withdrawal time (WT) of doxycycline in plasma and tissues [28]. WT is only estimated in edible tissues (muscle and skin) [29], but in this research was calculated in plasma and other tissues to identify the target organ where fish tissue depletes the least quickly. For the WT calculation, a tolerance limit based on the 95th percentile with a 95% level of confidence was used [28].

### 2.7. Statistical Analysis

Plasma and tissue concentrations of doxycycline were presented as mean ± standard deviation. The following formula was used to determine the temperature-dependent differences in pharmacokinetic parameters (10 versus 17 °C): [100 × (Value obtained at 17 °C−Value obtained at 10 °C)/Value obtained at 10 °C]. Both the ≤%(−) 25 and ≥% (+) 25 results were considered to be statistically significant [7,30].

## 3. Results

### 3.1. Pharmacokinetic Study

**Figure 1:**
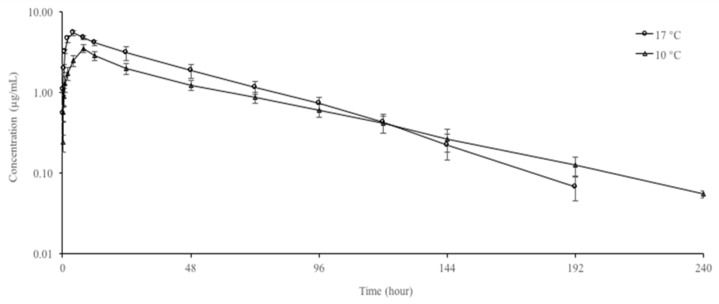
Semi-logarithmic plasma concentration–time curves of doxycycline following oral administration at a single dose of 20 mg/kg in rainbow trout (*Oncorhynchus mykiss*) at 10 and 17 °C.

The mean plasma concentrations of doxycycline at different water temperatures are shown in Figure 1. Doxycycline was measured up to 240 h at 10 °C and 192 h at 17 °C in plasma. At 10 and 17 °C, at the first observational time point (0.083 h), the plasma concentrations of doxycycline were 0.24 ± 0.06 and 0.55 ± 0.11 μg/mL, respectively. The plasma concentration of doxycycline dropped to 0.06 ± 0.10 μg/mL at 240 h at 10 °C and to 0.07 ± 0.02 μg/mL at 192 h at 17 °C. The pharmacokinetic parameters of doxycycline at different water temperatures are presented in Table 1. At 10 and 17 °C, the C_max_ and T_max_ of doxycycline were 3.48 and 5.50 μg/mL and 4 and 8 h, respectively. The increase in water temperature from 10 °C to 17 °C decreased t_1/2ʎz_, T_max_, CL/F, and V_darea_/F and increased C_max_ and AUC. The percentage AUC_extrap_ for both temperatures was less than 20%.

### 3.2. Residue Depletion Study and WT Estimation

The plasma and tissue concentrations of doxycycline in rainbow trout following daily oral administrations at 20 mg/kg for 5 days at 10 and 17 °C are presented in Table 2 and Table 3. The highest concentration of doxycycline in plasma and tissues was determined at 0.5 days after the last drug administration. Doxycycline was detected until 25 and 40 days in plasma and kidney, respectively, at both temperatures. In liver and muscle and skin, doxycycline was detected up to 50 and 35 days at 10 °C and up to 40 and 30 days at 17 °C, respectively. At both temperatures, the doxycycline’s concentration was obtained during liver > kidney > plasma > muscle and skin.

**Figure 2:**
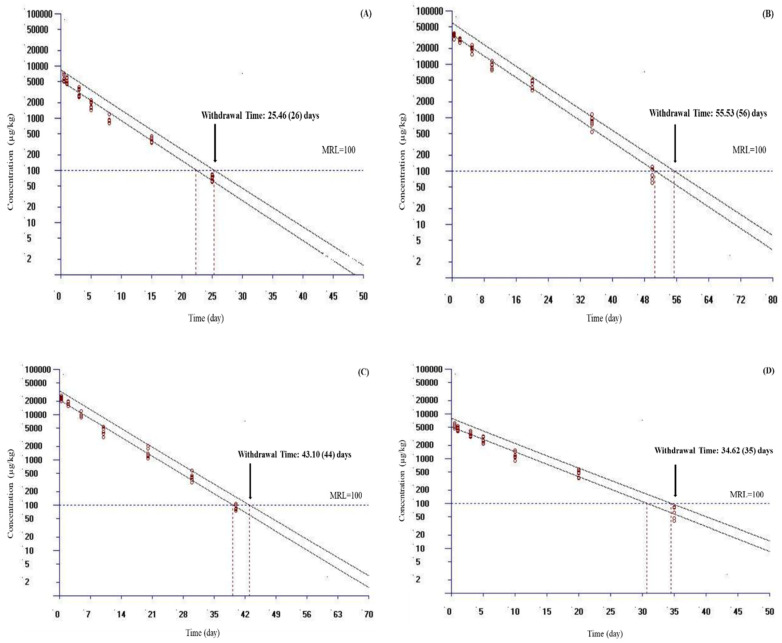
Calculated withdrawal times ((**A**) for plasma, (**B**) for liver, (**C**) for kidney, (**D**) for muscle and skin) of doxycycline in rainbow trout (*Oncorhynchus mykiss*) following daily oral administration at 20 mg/kg for 5 days at 10 °C. The withdrawal time was calculated by setting the maximum residue limits (MRL) at 100 μg/kg for Europe and China using the WT 1.4 software developed by the European Medicine Agency [28]. If the calculated withdrawal time was a fraction of a day, the estimated withdrawal time is provided in parenthesis, which was rounded up to the next day.

**Figure 3:**
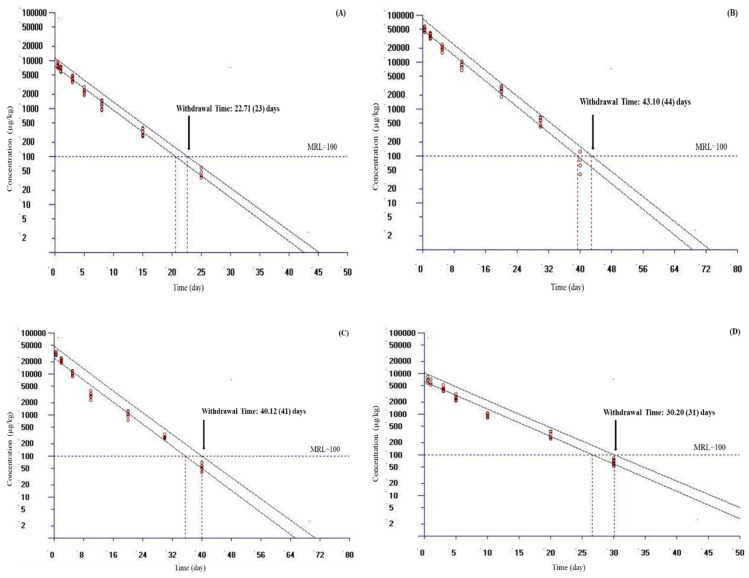
Calculated withdrawal times ((**A**) for plasma, (**B**) for liver, (**C**) for kidney, (**D**) for muscle and skin) of doxycycline in rainbow trout (*Oncorhynchus mykiss*) following daily oral administration at 20 mg/kg for 5 days at 17 °C. The withdrawal time was calculated by setting the maximum residue limits (MRL) at 100 μg/kg for Europe and China using the WT 1.4 software developed by European Medicine Agency [28]. If the calculated withdrawal time was a fraction of a day, the estimated withdrawal time is provided in parenthesis, which was rounded up to the next day.

The WT of doxycycline in plasma, liver, kidney, and muscle and skin was estimated using the WT 1.4 software program. WT values estimated according to MRL values are presented in Table 4. Because the WT 1.4 software program could analyze a dataset with a maximum of seven time points, only a portion of each dataset was used in the WT analysis in this study. In fish, the MRL value of doxycycline was reported only in muscle and skin tissue; this value was 100 μg/kg in Europe and China and 50 μg/kg in Japan [17,18]. At a water temperature of 10 °C, by setting the MRL at 100 μg/kg and considering a 95th percentile tolerance limit with a 95% confidence level for Europe and China, the calculated WT times were 56, 44, 35, and 26 days for liver, kidney, muscle and skin, and plasma, respectively (Figure 2). At the same temperature, by setting the MRL at 50 μg/kg and considering a 95th percentile tolerance limit with a 95% confidence level for Japan, the calculated WT times were 62, 49, 43, and 30 days for liver, kidney, muscle and skin, and plasma, respectively (Table 4). Compared with a temperature of 10 °C, the calculated MRL for plasma and other tissues was shorter at a water temperature of 17 °C (Figure 3).

For the WT calculation, a tolerance limit based on the 95th percentile with a 95% level of confidence was used. The calculated withdrawal time is provided in parenthesis; it was rounded up to the next day.

## 4. Discussion

The temperature-dependent plasma pharmacokinetics, tissue residues, and withdrawal times of doxycycline in rainbow trout were examined for the first time in this study. In countries such as China, Japan, India, and the Philippines, it is recommended to use doxycycline at a dose of 20 mg/kg for 3–5 days in fish [19]. No local or systemic side effects were observed in rainbow trout following single or multiple oral administrations of doxycycline at a dose of 20 mg/kg. In previous studies on various fish species, no side effects were observed following oral administration of 20 mg/kg doxycycline, either once or repeatedly [5,31,32]. Since it was stated that the optimal water temperature should be within the range of 9–20 °C in trout farming [33], temperatures of 10 and 17 °C were preferred in this study.

After oral administration of doxycycline to rainbow trout at 10 and 17 °C, t_1/2ʎz_, CL/F, and V_darea_/F were 28.87–41.72 h, 0.08–0.11 L/h/kg, and 3.42–6.82 L/kg, respectively. The t_1/2ʎz_, CL/F, and V_darea_/F of doxycycline after oral administration at different temperatures (18–28 °C) and different fish species (tilapia, catfish, grass carp) were reported as 5.81–77.2 h, 0.03–0.72 L/h/kg, and 0.87–5.81 L/kg, respectively [19,31,34]. The variability of doxycycline pharmacokinetic parameters in fish can vary due to changes in water temperature, fish species, drug, fish size, and drug administration.

At 10 and 17 °C, t_1/2ʎz_ of oral doxycycline to rainbow trout were 41.72 and 28.87 h, respectively. The increase in water temperature in trout significantly decreased the t_1/2ʎz_ of doxycycline. The increase in water temperature did not affect plasma t_1/2ʎz_ of doxycycline in grass carp, but shortened it in tissues such as liver, kidney, and muscle and skin in grass carp [19] and catfish [32]. Fish are poikilothermic animals; their body temperature is generally the same as the surrounding water temperature. The increase in water temperature in fish increases metabolic rates and cardiac outputs and decreases blood circulation time [35,36,37]. It has been stated that a temperature increase of 1 °C in fish provides a 10% increase in metabolic and excretion rates. In addition, in freshwater fish, decreased urine production at low water temperature results in decreased elimination of drugs [38]. In this study, the shortening of t_1/2ʎz_ with temperature may be due to the increase in metabolic rates and cardiac outputs and the acceleration of blood circulation.

After a single oral dose of doxycycline at 20 mg/kg, the C_max_ was estimated at 3.48 and 5.50 μg/mL at 10 and 17 °C, respectively, with a corresponding T_max_ of 8 and 4 h, respectively. The C_max_ and AUC values at 17 °C were higher than those at 10 °C. In grass carp, the increase from 18 °C to 24 °C in water temperature caused the high C_max_ (from 17.01 to 89.68 μg/mL) and AUC_0–last_ (from 308.66 to 644.78 h * μg/mL) [19]. In addition, the variation of C_max_, T_max_, and AUC values of marbofloxacin in crucian carp and enrofloxacin in turbot with temperature was similar to our study [39,40]. Water temperature improves cardiac output and blood circulation in fish, generating an increase in gastrointestinal system movement; increasing water temperature from 5 °C to 20 °C speeds up the emptying of gastric contents by 3–4 times [25,41]. The increase in C_max_ and AUC of doxycycline with an increase in temperature in rainbow trout may be due to increased absorption due to the above-mentioned reasons.

In this study, increasing the water temperature from 10 to 17 °C significantly decreased the CL/F (10 °C, 0.11 L/h/kg; 17 °C, 0.08 L/h/kg) and V_darea_/F (10 °C, 6.82 L/h/kg; 17 °C, 3.42 L/h/kg) values of doxycycline. Due to the increase in metabolic rates, cardiac outputs, and tissue blood supply with the increase in temperature, the values of CL/F and V_darea_/F were expected to increase; however, their decrease was surprising. Similar results were reported in the doxycycline study in grass carp [19]. Although few studies reported a slight decrease in CL/F at high temperature [38], most studies reported an increase in CL/F with temperature [22,42]. However, the equation CL/F = Dose/AUC_0–∞_ was used for the calculation of CL/F. Although the dose was the same at both temperatures, the AUC_0–∞_ value was 176.55 and 243.76 h*μg/mL at 10 and 17 °C, respectively. In this study, the increase in AUC_0–∞_ due to changes in metabolism or absorption with the effect of temperature may be the reason why the CL/F changed. However, to reveal the reason for the decrease in CL with an increase in temperature, it was necessary to determine the CL of doxycycline following IV administration in which the effect of absorption was not observed at temperatures of 10 and 17 °C. The calculation of V_darea_/F was based on the equation V_darea_/F = CL/F/λ_z_. The λ_z_ values for 10 and 17 °C were 0.017 and 0.024, respectively. In this study, the decrease in V_darea_/F with temperature may have been due to differences in CL/F and λ_z_.

In rainbow trout, after daily oral administration of 20 mg/kg for 5 days at 10 and 17 °C, the highest concentration for plasma, liver, kidney, and muscle and skin was reached at 0.5 days. At 10 °C, the highest concentrations for plasma, liver, kidney, and muscle and skin were 5.93 ± 0.82, 34.98 ± 2.82, 23.21 ± 2.22, and 5.31 ± 0.64 μg/mL, respectively. Doxycycline has a high lipophilicity and permeability that can result in high concentrations in various tissues after oral administration [43]. Compared with 10 °C, the C_max_ obtained in plasma and all tissues at 17 °C was higher. The higher C_max_ at 17 °C may have been related to increased absorption due to the high temperature. Doxycycline concentrations in the liver and kidney were much greater than in the plasma. Similar findings have been observed in the research on catfish and grass carp [5,32]. Doxycycline was extensively (about 40%) metabolized in the liver and most of it was excreted in the feces via bile and intestinal secretion [14]. In some studies, it has been reported that 35–60% of doxycycline was excreted in the urine [44]. The reason for the high concentration of doxycycline in the liver and kidney may be because they are metabolic and elimination organs.

In this research, the WT 1.4 software program was used to determine WT values in plasma and tissues. In this study, muscle and skin was preferred because they are the main edible tissues; liver and kidney are the main organs of metabolism and elimination [19]. Many countries have set an MRL from food products to protect human health. The MRL value of doxycycline was determined only for muscle and skin tissue in fish. The muscle and skin tissue MRL has been reported as 100 μg/kg in Europe and China and as 50 μg/kg in Japan [17,18]. At a water temperature of 10 °C, by setting the MRL at 100 μg/kg for Europe and China, the calculated WT times were 55.53, 43.10, 34.62, and 25.46 days for liver, kidney, muscle and skin, and plasma, respectively. At the same temperature, by setting the MRL at 50 μg/kg for Japan, the calculated WT times were 61.65, 48.31, 40.20, and 29.52 days for liver, kidney, muscle and skin, and plasma, respectively. Compared with at 10 °C, the calculated MRL for plasma and other tissues was shorter at a water temperature of 17 °C. These results showed that the increase in water temperature shortened the WT. In addition, these data were also compatible with plasma and tissue t_1/2ʎz_ of doxycycline determined at 10 and 17 °C. After oral administration of 20 mg/kg doxycycline for 3 days in grass carp at 24 °C, the WT (100 μg/kg for Europe and China) values for plasma, liver, kidney, and muscle and skin were reported as 25, 56, 42, and 41 days, respectively [5]. The difference in doxycycline WT value between rainbow trout and grass carp may be due to different fish species, dosage regimen, and temperature.

## 5. Conclusions

It can be concluded that temperature has profound effects on the pharmacokinetic behavior of doxycycline. Following the increment of water temperature from 10 to 17 °C, t_1/2ʎz_, T_max_, CL/F, and V_darea_/F of doxycycline decreased and plasma concentrations increased. In this study, since several important pharmacokinetic parameters changed with temperature, a temperature-dependent dosing regimen of doxycycline might be necessary. To ensure that edible tissues (muscle and skin) are safe for consumption, a WT of doxycycline at 10 and 17 °C after oral administration at 20 mg/kg dose for 5 days in rainbow trout should not be less than 35 and 31 days, respectively, for Europe and China and 41 and 35 days, respectively, for Japan.

## Figures and Tables

**Table 1:** Pharmacokinetic parameters of doxycycline following oral administration at a single dose of 20 mg/kg in rainbow trout (*Oncorhynchus mykiss*) at 10 and 17 °C.

Parameters	10 °C	17 °C	%GD *
t_1/2ʎz_ (h)	41.72	28.87	−30.81
AUC_0–last_ (h * μg/mL)	173.23	240.96	39.10
AUC_0–∞_ (h * μg/mL)	176.55	243.76	38.07
AUC_extrap_ (%)	1.88	1.20	
MRT_0–∞_ (h)	60.42	46.01	−23.85
CL/F (L/h/kg)	0.11	0.08	−27.57
V_darea_/F (L/kg)	6.82	3.42	−49.89
C_max_ (μg/mL)	3.48	5.50	58.05
T_max_ (h)	8	4	−50.00

* Refers to the percentage (%) difference between 17 °C administration and 10 °C administration. t_1/2λz_—terminal elimination half-life; AUC—area under the plasma concentration–time curve; AUC_extrap_ %—area under the plasma concentration–time curve extrapolated from t_last_ to ∞ in % of the total AUC; MRT—mean residence time; CL/F—total clearance; V_darea_/F—apparent volume of distribution, C_max_—peak plasma concentration; T_max_—time to reach C_max_.

**Table 2:** Plasma and tissue concentrations of doxycycline in rainbow trout (*Oncorhynchus mykiss*) following daily oral administration at 20 mg/kg for 5 days at 10 °C (n = 6, mean ± SD).

Time (Days)	Plasma (µg/mL)	Liver (µg/g)	Kidney (µg/g)	Muscle and Skin (µg/g)
0.04	5.06 ± 0.82	28.40 ± 2.35	18.63 ± 2.28	4.35 ± 0.75
0.5	5.93 ± 0.82	34.98 ± 2.82	23.21 ± 2.22	5.31 ± 0.64
1	5.13 ± 0.63	32.60 ± 1.68	20.25 ± 1.86	4.69 ± 0.54
2	4.28 ± 0.56	29.09 ± 1.94	18.02 ± 1.72	4.08 ± 0.48
3	3.18 ± 0.60	25.91 ± 2.78	15.13 ± 1.00	3.55 ± 0.44
4	2.39 ± 0.34	22.54 ± 2.22	12.22 ± 1.34	3.08 ± 0.44
5	1.82 ± 0.29	19.49 ± 2.45	9.79 ± 1.27	2.57 ± 0.41
6	1.36 ± 0.22	16.17 ± 2.39	7.61 ± 1.24	2.06 ± 0.32
8	0.99 ± 0.17	12.89 ± 2.03	5.71 ± 0.96	1.61 ± 0.29
10	0.72 ± 0.13	9.86 ± 1.59	4.20 ± 0.83	1.22 ± 0.23
15	0.38 ± 0.05	6.63 ± 1.04	2.54 ± 0.82	0.78 ± 0.13
20	0.15 ± 0.03	4.07 ± 0.77	1.46 ± 0.37	0.47 ± 0.09
25	0.07 ± 0.01	2.53 ± 0.60	0.81 ± 0.15	0.25 ± 0.07
30	LLOQ	1.53 ± 0.34	0.43 ± 0.09	0.12 ± 0.03
35	LLOQ	0.85 ± 0.20	0.22 ± 0.03	0.06 ± 0.02
40	LLOQ	0.44 ± 0.16	0.09 ± 0.01	LLOQ
50	LLOQ	0.09 ± 0.02	LLOQ	LLOQ

<LLOQ—below lower limit of quantification of 0.04 μg/mL or μg/g.

**Table 3:** Plasma and tissue concentrations of doxycycline in rainbow trout (*Oncorhynchus mykiss*) following daily oral administration at 20 mg/kg for 5 days at 17 °C (n = 6, mean ± SD).

Time (Days)	Plasma (µg/mL)	Liver (µg/g)	Kidney (µg/g)	Muscle and Skin (µg/g)
0.04	6.84 ± 0.80	42.89 ± 5.61	22.66 ± 2.98	5.83 ± 0.91
0.5	8.06 ± 0.87	51.47 ± 5.80	32.01 ± 2.75	7.13 ± 0.97
1	6.76 ± 0.64	44.06 ± 4.85	27.44 ± 1.89	6.19 ± 0.83
2	5.59 ± 0.61	37.06 ± 4.69	21.67 ± 2.35	5.14 ± 0.73
3	4.26 ± 0.51	30.13 ± 3.50	17.33 ± 1.81	4.24 ± 0.56
4	3.19 ± 0.50	24.71 ± 2.68	13.69 ± 1.84	3.29 ± 0.47
5	2.30 ± 0.31	19.86 ± 2.60	10.12 ± 1.47	2.52 ± 0.37
6	1.71 ± 0.31	15.81 ± 2.35	6.82 ± 1.06	1.84 ± 0.23
8	1.20 ± 0.22	11.68 ± 1.43	4.66 ± 0.87	1.33 ± 0.19
10	0.76 ± 0.17	8.59 ± 1.22	3.19 ± 0.66	0.94 ± 0.10
15	0.32 ± 0.06	4.80 ± 0.97	1.68 ± 0.26	0.54 ± 0.09
20	0.13 ± 0.02	2.51 ± 0.42	1.04 ± 0.18	0.31 ± 0.05
25	0.04 ± 0.01	1.15 ± 0.19	0.55 ± 0.09	0.16 ± 0.03
30	LLOQ	0.56 ± 0.10	0.29 ± 0.03	0.07 ± 0.01
35	LLOQ	0.25 ± 0.05	0.14 ± 0.02	LLOQ
40	LLOQ	0.08 ± 0.03	0.05 ± 0.01	LLOQ
50	LLOQ	LLOQ	LLOQ	LLOQ

<LLOQ—below lower limit of quantification of 0.04 μg/mL or μg/g.

**Table 4:** Calculated withdrawal times of doxycycline in rainbow trout (*Oncorhynchus mykiss*) following daily oral administration at 20 mg/kg for 5 days at 10 and 17 °C.

Plasma and Tissues	10 °C		17 °C	
	Europe and China (Days)	Japan (Days)	Europe and China (Days)	Japan (Days)
Plasma	25.46 (26)	29.52 (30)	22.71 (23)	26.08 (27)
Liver	55.53 (56)	61.65 (62)	43.10 (44)	47.61 (48)
Kidney	43.10 (44)	48.31 (49)	40.12 (41)	44.73 (45)
Muscle and skin	34.62 (35)	42.20 (43)	30.20 (31)	34.80 (35)

## Data Availability

The data presented in this study are available on request from the corresponding author.
